# The Validity of the Theory of Planned Behaviour for Understanding People’s Beliefs and Intentions toward Reusing Medicines

**DOI:** 10.3390/pharmacy9010058

**Published:** 2021-03-09

**Authors:** Hamza Alhamad, Parastou Donyai

**Affiliations:** 1 Department of Pharmacy, University of Reading, Reading RG6 6DZ, UK; 2 Department of Pharmacy, Zarqa University, P.O. Box 132222, Zarqa 13132, Jordan

**Keywords:** medicines reuse, medication waste, psychological theories, theory of planned behaviour, people’s belief, people’s intentions

## Abstract

Background: many factors can impact a person’s behaviour. When the behaviour is subject to prediction, these factors can include, for example, the perceived advantages and disadvantages of performing the behaviour, normative beliefs, and whether the behaviour is thought to be achievable. This paper examines intentions to engage in medicines reuse, i.e., to accept medicines that are returned unused to a pharmacy to be reused. The paper aims to outline the validity of the Theory of Planned Behaviour (TPB) for understanding people’s intentions to engage in medicines reuse by examining this against other long-standing health-related psychological theories of behavioural change. Thus, the Health Belief Model (HBM), Protection Motivation Theory (PMT), Trans-Theoretical Model of Health Behaviour Change (TTM/SoC), Theory of Reasoned Action (TRA), and TPB are examined for their application in the study of medicines reuse. Discussion: the HBM, PMT, TTM/SoC, TRA, and TPB were assessed for their relevance to examining medicines reuse as a behaviour. The validity of the TPB was justified for the development of a Medication Reuse Questionnaire (MRQ) to explore people’s beliefs and intention toward reusing medicines. Conclusion: TPB has been widely used inside and outside of health-related research and it was found to have more accurately defined constructs, making it helpful in studying medicines reuse behaviour.

## 1. Introduction

A multitude of factors can influence people’s behaviour. The behaviour of interest here is whether people will accept medicines that are returned unused to a pharmacy for their own use (i.e., take part in medicines reuse). The influencing factors for medicines reuse could include, for example, the perceived advantages and disadvantages of performing the behaviour, views about the therapeutic classes and safety [1,2], and storage conditions [3] of returned unused medicines, and social pressure or normative belief regarding reusing medicines. Understanding the precise nature and significance of these factors is not straightforward, but could be explored using psychological theory. Additionally, as well as providing a generalisable organising framework for studying and predicting potentially foreseeable behaviour [4], psychological theory can also provide a mechanism for changing people’s behaviour, which is of added interest to health practitioners and policy-makers.

Arguably, then, the application of a framework to study people’s thoughts and behavioural responses to medicines reuse could not only help to explain, but also enable relevant stakeholders to predict and influence medicines reuse behaviour [5]. However, while there are many different and overlapping health-related psychological theories and models available in the literature [6,7,8], none have been examined for applicability in relation to medicines reuse until our own research. The lack of guidance regarding how to select a suitable theory for a particular research interest [4,9] meant this was not a straightforward task. One suggestion to improve the selection of theory across relevant disciplines is to consider all of those psychological theories that could be of potential use in informing public health questions, and then narrow down according to the particular behaviour, population, and context of the research [10]. This review aims to do that by providing an overview of common health-related behavioural change theories, justifying the selection of a particular theory and then briefly describing steps that were required to manage the development of a Medication Reuse Questionnaire (MRQ) to explore people’s beliefs and intention toward reusing medicines based on the selected theory. An argument is made for the validity of the Theory of Planned Behaviour (TPB) to predict *medicines reuse* behaviour. Subsequent to the work described in this paper, the TPB was successfully applied to understand people’s conceptualization of this behaviour [11] and model and measure their intention to reuse medicines in the future [12]. 

## 2. Overview of the Common Health-Related Behavioural Change Theories 

Many psychological theories and models attempt to explain the relationship between people’s thoughts, beliefs, decisions, and behaviours; however, not all are unconditionally helpful, health-related, or, in fact, evidenced-based [13]. Additionally, numerous theories have been criticised based on their (in)effectiveness and lack of predictive power, unclear construct development, and lack of guidelines on how exactly they could be used to measure behaviour or intention toward a behaviour [13]. The more common and frequently-used health-related behavioural change theories that are potentially relevant to medicines reuse as a behaviour are reviewed [7,8,14]; these include, the Health Belief Model (HBM), Protection Motivation Theory (PMT), Trans-Theoretical Model of Health Behaviour Change (TTM/SoC), Theory of Reasoned Action (TRA), and the Theory of Planned Behaviour (TPB). The majority of these theories focus on behaviours that relate directly to health, e.g., smoking cessation, but there is also a precedence for applying these theories to other behaviours, such as those that are linked to the environment and waste reduction, meaning that these theories could potentially be relevant to studying medicines reuse as a behaviour. 

### 2.1. Health Belief Model (HBM)

**Figure 1:**
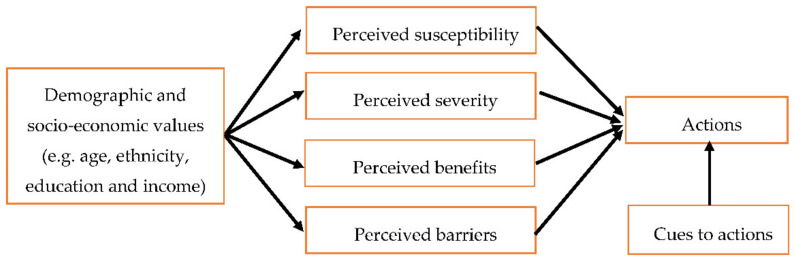
A graphical representations of the Health Belief Model (HBM) [8].

The Health Belief Model (HBM) is one of the earliest psychological health models, developed in the 1950s to predict preventive health behaviours and the behavioural reaction to treatment in acutely and chronically ill patients [15]. Over recent years, the HBM has been used to try and improve many health-related interventions by predicting a wide variety of health-related behaviours [8,16]. The HBM constructs consist of; *perceived susceptibility,* including a person’s perception regarding the risk of the (maladaptive) health behaviour (e.g., susceptibility to lung cancer because of a behaviour such as smoking); *perceived severity* of the threat to health via the behaviour (e.g., severity of lung cancer as an illness); *perceived benefits* from taking action to change the behaviour (e.g., stopping smoking will save money and reduce my illness); *perceived barriers* towards the behaviour or the costs that are involved in performing the behaviour (e.g., stopping smoking will make me irritable); *cues to actions,* which might be internal (e.g., family member illness due to smoking) or external (e.g., television news and reports about the ill effects of smoking); and, *demographics and socio-economic values* (e.g., age, ethnicity, education, and income) [7,8]. Each of the individual constructs or in combination can theoretically be used to predict the likelihood that the behaviour change will occur (Figure 1). Yet, the HBM has received many criticisms, including that it has weak predictive power in most areas of health-related behaviour [7,14], poor construct definition, and that other core psychological factors are missing from the model, including environmental or economic issues that might also impact behaviours [7,14]. Variables, such as intentions to carry out a specific behaviour and the influence of social pressure, which can be highly predictive of behaviour, are also absent from the HBM [17]. Importantly, the HBM does not include clear guidelines on how its variables might be combined and operationalised, especially the constructs of benefits and barriers [14]. The literature on the usefulness of the HBM is contradictory, but studies utilising this model or different aspects of the model’s constructs report it to predict some health-related behaviours, such as taking part in screening for hypertension, screening for cervical cancer, genetic screening, exercise behaviour, decreased alcohol use, changes in diet, and smoking cessation [7,8]. The HBM was considered here, because of its prevalence in health psychology research and because medicines reuse could arguably be perceived as a preventive behaviour (e.g., helping to prevent environmental waste through reuse could improve health indirectly). Indeed, some of the constructs of the HBM could be seen as relatable to medicines reuse behaviour (e.g., perceived benefits, perceived barriers, and cues to action). However, because medicines reuse is not a health condition or behaviour that can directly impact on a person’s health, some of the other constructs of the HBM cannot be judged as applicable at all (e.g., perceived susceptibility and perceived severity), which renders this theory ineffective for our purposes. To explain, in the HBM the construct, perceived susceptibility relates to a person’s perception regarding the risk of the maladaptive behaviour and perceived severity relates to how bad this health threat would be. However, for our purposes, medicines reuse would be defined as the ‘favoured’ behaviour, which would make these constructs redundant as the act of reusing medicines is not directly *preventing* a condition. 

### 2.2. Protection Motivation Theory (PMT) 

**Figure 2:**
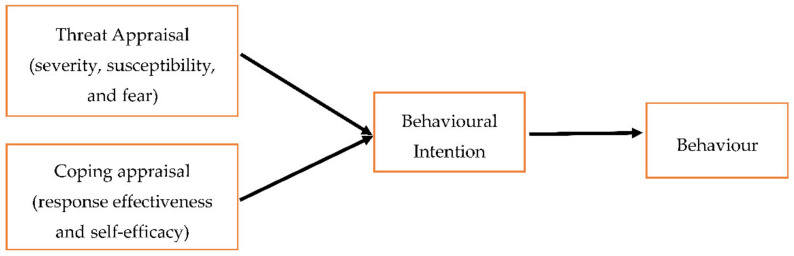
A graphical representation of the Protection Motivation Theory (PMT) [8].

The Protection Motivation Theory (PMT) is considered to be a revised version and expansion to HBM to include additional constructs. According to PMT, the primary determinant to carry out a health-related behaviour is protection motivation or intention to carry out the behaviour, and the behaviour change may be achieved by engaging with an individual’s fears [18]. Protection motivation is determined by *threat appraisal* and the *coping appraisal process*. *Threat appraisal* is referred to as a cognitive process that the individual uses to assess the level of threat (including severity, susceptibility, and fear), while the *coping appraisal process* refers to the individual’s assessment of their ability to carry out risk preventive behaviour which influences the protection motivation (including response effectiveness and self-efficacy) (Figure 2) [19]. Together, the outcome of the appraisal processes is classified into either adaptive (adopting health behaviour) or maladaptive responses (avoidance or denial of health threat) [8,17]. The PMT has been successfully applied to predict several health behaviours and it is less widely criticised when compared to HBM [20]. Nonetheless, PMT does not account for habitual behaviours (e.g., brushing teeth), nor does it include social (e.g., what others think/do) and environmental factors (e.g., opportunities to exercise or eat appropriately at work) [8]. However, the main reason it lacks utility for studying medicines reuse behaviour, similar to the HBM, is because medicines reuse does not pose a direct health threat to individuals, which means that the main constructs (e.g., threat appraisal) are not valid for application to medicines reuse.

### 2.3. Trans-Theoretical Model of Behaviour Change or Stages of Change (TTM/SoC) 

**Figure 3:**
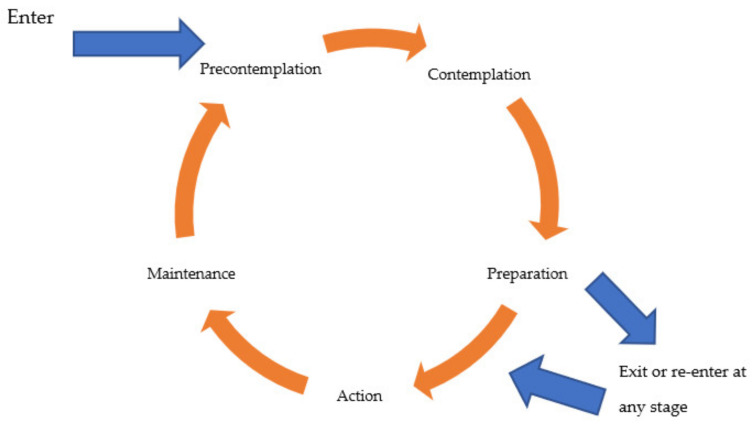
A graphical representation of the Trans-Theoretical Model of Behaviour Change or Stages of Change (TTM/SoC).

The TTM/SoC was specifically designed to facilitate behavioural change [7]. TTM/SoC provides information regarding different target groups and how they should be approached. It has received empirical support with regard to different health-related behaviours and is a widely used cognitive model [7]. TTM/SoC divides individuals into five stages that represent different levels of motivational willingness to change their behaviour. These stages were first developed about smoking and include; *pre-contemplation* (e.g., the person might think “I am happy being a smoker and intend to continue”), *contemplation* (e.g., “recently, I have been coughing a lot, maybe I should think about stopping smoking”), *preparation* (e.g., “I will buy fewer cigarettes”), *action* (e.g., “I have stopped smoking”), and maintenance (e.g., “I have stopped smoking for five months now”) [8,21]. The model allows for people to exit and re-enter, including cases of relapse. In some versions of the TTM/SoC, the final stage, *termination,* is added. In this stage, the new behaviour is seen as being entirely determined after a period of five or more years (see Figure 3) [7]. 

The transition between stages is thought of as being controlled by self-efficacy and decisional balance constructs. Self-efficacy (which is also included in the HBM and TPB) is expected to increase as individuals move toward action and maintenance stages. Decisional balance measures the individual’s relative balancing of the advantages and disadvantages of changes that combine to form a decision. This balance between advantages and disadvantages mainly depends on which stage of change the individual is in [22]. There are many criticisms regarding the complexity of the TTM/SoC model, how distinct the stages really are, and whether an individual would actually move through each stage. Moreover, movement between the stages can occur so quickly as to make the distinction between stages less valuable [8]. Consequently, the TTM/SoC model is less clear on how individuals change or the reasons some change more efficiently than others [21]. Another criticism of the TTM/SoC model is that the effectiveness of a stage-based intervention differs based on the behaviour [23]. Some have called for a more coherent definition of the stages in the TTM/SoC model, as well as some level of standardisation [24]. 

Having considered the TTM/SoC model and its potential advantages and disadvantages, its use for studying medicines reuse was discounted, as explained here. Because the practice of medicines reuse does not currently take place in the UK, there was no experience of this behaviour to draw on in order to delineate the difference between the distinct stages unique to the TTM/SoC. Thus, neither an interview study nor an observational study could have possibly elicited relevant information against these very specific constructs that rely on actual experience. 

### 2.4. The Theory of Reasoned Action (TRA) and the Theory of Planned Behaviour (TPB) 

**Figure 4:**
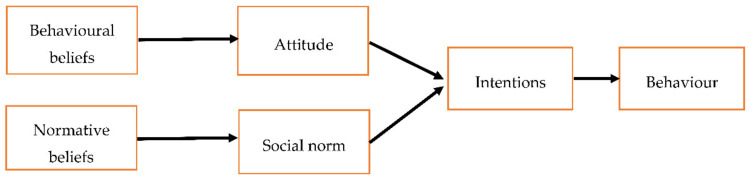
A graphical representation of the Theory of Reasoned Action (TRA) model [26].

Fishbein and Ajzen developed the TRA in 1967 to examine the relationship between beliefs, attitudes, intentions, and behaviour [25]. The TRA assumes that an individual’s intention to perform a behaviour is the most proximal antecedent of that behaviour. Individuals’ intentions are, in turn, influenced by their attitudes toward performing the behaviour and the subjective/social norms relating to behavioural performance (Figure 4). Therefore, the TRA is a model in which the individual is positioned within the social context [8]. Ajzen later expanded the TRA to develop the TPB by taking account of what people believe stops or facilitates their behaviour.

**Figure 5:**
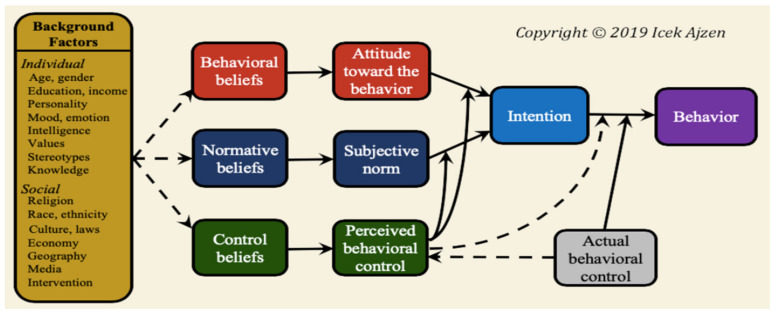
A graphical presentation of the Theory of Planned Behaviour Model (TPB) with background factors [30].

In the TPB, Ajzen attempted to evolve and extend the TRA by adding the perceived behavioural control (PBC) construct. PBC is a construct describing the factors that control the individual’s decision to carry out the behaviour. PBC is considered to be representative for actual control, as it is expected to moderate the effect of intention on behaviour [26]. The intention to perform the behaviour is considered the key determinant of behaviour in the TPB [6]. Here, the stronger the intentions to engage in behaviour, the more likely behaviour will be performed [27]. The TPB proposes a framework in which cognitions (i.e., behavioural, normative, and control beliefs) and broader constructs (i.e., attitude toward the behaviour, subjective norm, and perceived behavioural control) influence behaviour [28] via intentions [8,29]. Moreover, in this model, the PBC construct itself could predict behaviour without the effect of intention [8]. The TPB could make the following predictions if TPB is applied to medication reuse: if a person believes that reusing their medicines would benefit the economy and environment, and would be useful to their own health (i.e., attitude toward the behaviour), that essential people in their life would like them to reuse medicine (i.e., subjective norm), and that they have the ability to reuse medicines in the future after evaluating the internal and external factors that allow or preclude medicine reuse (i.e., PBC), then this could predict a high intention to reuse medicines in the future. On the face of it, then, the constructs of the TPB could all be relevant in determining medicines reuse behaviour, albeit via the intention construct. Additionally, Ajzen recognised the importance of demographics variables and later added the background factors to the TPB [30,31]. The background factors impact intentions and behaviour indirectly by affecting behavioural, normative, and/or control beliefs [30,31]. That is, background factors can supply useful information regarding possible precursors of behavioural, normative, and control beliefs (Figure 5).

## 3. Discussion

The main focus of this discussion is to assess, support, and argue for the validity of the TPB to predict people’s behavioural beliefs and their intentions to reuse medicines in the future. The steps that are used to develop a TPB Medication Reuse Questionnaire (MRQ) to explore people’s beliefs and intention toward reusing medicines are also described.

### 3.1. The TPB Compared to the TRA, HBM, PMT, and TTM/SoC

The TPB, TRA, HBM, PMT, and TTM/SoC are particular models that have a number of constructs relating to behaviour in common [14,15,21]. The construct commonalities involve components relating to how individuals balance the perceived costs and benefits of alternative behaviours; beliefs about others’ expectations and values relating to health behaviours; the formation of intentions to act (except for the HBM); and, individuals’ self-efficacy perceptions regarding taking behavioural action (except for the TRA) [7,32,33]. For example, self-efficacy, perceived barriers and benefits described within the HBM, could be seen as being very similar to control beliefs and behavioural beliefs described in the TPB [34]. However, some of these constructs are only unique to a particular theory [13,32,33]. For example, the perceived threat construct of HBM described as perceived seriousness and perceived susceptibility to the illness does not appear in the TRA, TPB, and TTM/SoC models. This can be seen as an advantage in which the perceived threat construct can describe the consequences of reusing medicines that have been tampered with or contaminated. Moreover, the HBM includes objective demographics and cue to action constructs that are not included in the TRA, TPB, and TTM/SoC models, which can be seen as a another potential advantage [7]. However, the evidence indicates that the HBM’s objective demographics and cue to action constructs, although perceived as potential strengths, have not been effectively used in practice [7]. In any case, the HBM is more health-behaviour focused as compared to the TRA and TPB, which are designed to be applicable to more general behaviours; thus, the TRA and the TPB can be applied outside as well as inside the health discipline [5,6,7,10,16,26,32,34,35,36,37,38,39,40,41,42,43,44,45,46,47,48]. The combination of TPB and the TTM/SoC has been tested with good results. For instance, TPB adds to our understanding of what motivates the behaviour, whereas TTM/SoC provides information regarding different target groups and how they should be approached. TTM/SoC has also received empirical support with regard to different health-related behaviours and it is a widely used cognitive mode. The TRA and the TPB have identical attitudinal and social norm constructs in common; however, the TPB, contains a PBC construct relating to control related beliefs and self-efficacy [26,27,31]. With the help of the revised TPB, it becomes possible to examine why a given background factor influences, or fails to influence, behaviour by following its effects through the more proximal antecedents of the behaviour [30,31]. The TRA and the TPB have fewer, but more accurately defined, constructs and they are mathematically better specified than the HBM and the TTM/SoC models. This promotes the adequacy and consistency of the use of TRA and TPB [7]. The TPB is more successful in predicting behaviour and it has been widely used inside and outside health-related research [6,7,21,26,32,35,36,37,38,39,40,41,42,43,44,45,46,47,48]. There is meta-analytic and systematic review evidence that the predictive performance of both the TRA and the TPB is superior to the HBM [7]. Moreover, the additional constructs that are contained in the TPB allow it to have a more significant predictive percentage of the overall behavioural variance than the TRA [7]. The available evidence suggests that the application of the TPB in countries, such as USA and UK, can predict around 20–30% of the observed variance of health behaviours [7]. Additionally, there is a strong correlation between behaviour and both attitudes towards the behaviour and PBC constructs of TPB [7]. However, the correlation between behaviour and subjective norms is less and is sometimes referred to as a weak correlation [21]. The issue of the weak correlation was argued to be probably methodological, as a small number of studies that measured subjective norms fairly reported strong relationships with behaviour [6,21].

### 3.2. Support for the Application of TPB to Predicts People’s Behaviour and Intention towards Reusing Medicines

The TPB is a framework that has been widely applied in a variety of domains for predicting and explaining behaviour and increasingly for conducting behaviour change interventions [27,28,49]. There have been several reviews and meta-analyses describing the generalisability of the TPB in different behavioural domains and its effectiveness to predict a range of health behaviours [6,7,8]. The generalisability of TPB-based interventions is illustrated in a recent meta-analysis [28]. The studies reviewed were concerned with reducing alcohol consumption [50,51], smoking cessation [36,47], predicting adherence to medicines [48,52], promoting hand hygiene [53], nutrition-related intervention, such as promoting whole-grain foods by dieticians [37], and food safety [38], physical activity [39], and weight control [40,54], sexual behaviour related interventions, such as promoting safer sex practices [41,55,56], traffic-related interventions, such as promoting school-age cyclists to wear safety helmets [42], and promoting drivers’ compliance with speed limits [43], and work-related interventions, such as promoting work health and safety [57]. In addition to the above, TPB-based interventions have been applied in other domains, such as environment and sustainability [44,58,59], reuse [60], recycling [35,45], and intention to donate to charity [61]. The effectiveness of TPB-based interventions in predicting behavioural changes is illustrated in the quantitative meta-analysis review of 185 independent studies published up to the end of 1997, where it was found that across all behaviours, the average multiple correlations of intention and PBC with behaviour was 0.52, accounting for 27% of the variance, and the average multiple correlations of attitude, subjective norm, and PBC with intention was 0.63, accounting for 39% of the variance [6]. Finally, the correlation between subjective norms and the behavioural intention was found to be weaker than those between attitudes and the behavioural intention and between PBC and behavioural intention [6]. In 1991, Ajzen conducted a review of 16 studies involving the TPB to examine the effectiveness of TPB-based interventions in predicting the behavioural changes and found that attitude, subjective norm, and PBC accounted for a significant amount (20% to 78%) of variance in behavioural intention [27]. The multiple correlations between behavioural intention and its three predictors (i.e., attitude, subjective norm, and PBC) ranged from (0.43 to 0.94), with an average correlation of 0.71. Moreover, Ajzen added that PBC, together with intention, were significant predictors of behaviour, with the average multiple correlations being 0.51 [27]. Finally, in a review of 56 studies, the variance in behavioural intention that was explained by TPB constructs was 40.9%, and PBC was a significant predictor of behavioural intention in 85.5% of health-related studies, followed by attitude (81.5%) and subjective norm (74.4%) [46]. PBC contributed a mean additional 13% of variance to the prediction of behavioural intentions, over and above the attitude and subjective norm constructs, and 12% to the prediction of behaviour [46]. The PBC figures that were reported in this review [46] were higher than those reported by the study of Armitage and Conner [6]. Subjective norm was a strong predictor of the behaviour in the study by Godin and Kok [46] as compared to the Armitage and Conner study [6], which was reported to be a weak predictor of the behaviour. Ajzen stated that intentions are heavily influenced by personal factors, such as attitudes and perceived behavioural control [27]; however, Ajzen recommends the inclusion of injunctive (i.e., expectation or subjective probability that a referent individual or group, such as friends, family, spouse, coworkers, one’s physician, or supervisor approves or disapproves of performing the behaviour under consideration) and descriptive (i.e., beliefs as to whether important others themselves perform the behaviour) norms as a solution to improve the correlation between subjective norm and intention [31,62]. These reviews and meta-analyses support the empirical applicability and popularity of TPB, and demonstrate that TPB, overall, is quite a successful model in explaining and predicting behavioural intentions and actual behaviours. Despite the addition of PBC to the TPB, other variables that may control the actual behaviour, such as desire, need, and emotion, are still lacking [31,63]. These factors may affect the actual behaviour, regardless of the expressed attitude [31,63]. For example, a person may have a positive attitude towards reusing medicine, but do not need, or do not have, a desire to reuse medicine. Based on the above strength and limitation, TPB was chosen to be applied to understand people’s beliefs and intentions to reuse medicines in the future.

### 3.3. Steps to Manage the Development of a TPB Medication Reuse Questionnaire (MRQ) to Explore People’s Beliefs and Intention toward Reusing Medicines

When the TPB as a psychological framework is applied, specific steps are followed to enhance the validity of the research. These steps are according to the recommendations made by Francis and Ajzen [29,64]. The first step would then be to define medicines reuse as behaviour an select the population of interest. The TACT principle is used, by which the behaviour is defined according to target, action, context, and time. For example, for the behaviour “capturing people’s beliefs and intention to reuse medication that is returned to pharmacies by another patient”, the target is people in general, the action is their beliefs and intentions to reuse medication, the context is reusing medication that is returned to pharmacies by another patient, and the time is in the future. Medicine reuse as behaviour was primarily defined as “accepting prescribed medication with more than six months of shelf-life remaining that, as verified by a pharmacist, had been kept untampered for less than three months, under normal storage conditions and in an original sealed blister pack, by another patient before being returned to a community pharmacy” [11]. A sample of the population of interest for an elicitation (i.e., qualitative) study then needs to be determined. The sample size for an elicitation study is aimed to be between 15–20 participants. The second step is to complete the elicitation study to develop the indirect measures (behavioural beliefs, normative beliefs, and control beliefs) for all of the predictor constructs of the TPB (attitude, subjective norms, and PBC). An elicitation study was indeed completed with a sample of 19 participants that were interviewed face to face. Themes obtained from the elicitation study were classified according to the TPB constructs and they were used to develop the questions related to the indirect measures of the TPB [11]. The third step was to develop the MRQ. The MRQ questions are of three types; first, the questions developed from the elicitation study that are related to the indirect measures of TPB, second, the question related to the direct measures of TPB, and third, the questions related to the background factors that are important and related to medicines reuse. All of the MRQ questions were indeed developed according to Francis and Ajzen recommendations [29,64]. The fourth step was to pilot and validate the MRQ. Validity and reliability testing were also applied. Content validity is applied by asking cognitive questions, and questions at the end of the interview, such as: are any items difficult to answer or ambiguous; does the questionnaire feel too repetitive; does it feel too long; does it feel superficial; and, are there any annoying features of the wording or formatting? Reliability testing was applied, including internal consistency for the direct measures of TPB and test-retest reliability for the indirect measures of the TPB [12]. Fifth, Confirmatory Factory Analysis (CFA) was applied to the MRQ in order to confirm that the questions measuring each construct are considered indicators of the same latent variable; and, the TPB model in which the attitude, subjective norm, PBC, and intention items are treated, as assessing separate constructs is superior to a model in which all questions are considered to measure the same underlying construct [12]. The sixth step was to use the MRQ to capture the representative views regarding people’s beliefs and willingness to reuse medicines in the future [12]. The data about the development, validation, and evaluation of a TPB model used to predict medicines reuse behavioural intentions were successfully used to understand people’s intention to reuse medicines in the future [12].

## 4. Conclusions

This review summarised the common and frequent health-related behavioural change theories that might be potentially relevant to medicines reuse behaviour. The need for the psychological framework was described and the rationale presented for selecting TPB as an appropriate theory to develop the MRQ to explore people’s beliefs and intention toward reusing medicines in the future. The TPB has been widely used inside and outside health-related research, and it has been found to have more accurately defined constructs and be better mathematically specified than the HBM and the TTM/SoC. The TPB was found to be more useful in studying medicines reuse behaviour because of its wider use outside of health behaviours and the apparent relevance of its constructs. The theory has since been applied in both an elicitation study as well as a large-scale questionnaire study measuring people’s attitudes to medication reuse.
